# Early Fault Detection of Rolling Bearings Based on Time-Varying Filtering Empirical Mode Decomposition and Adaptive Multipoint Optimal Minimum Entropy Deconvolution Adjusted

**DOI:** 10.3390/e25101452

**Published:** 2023-10-16

**Authors:** Shuo Song, Wenbo Wang

**Affiliations:** Hubei Province Key Laboratory of System Science in Metallurgical Process, Wuhan University of Science and Technology, Wuhan 430081, China; songshuo@wust.edu.cn

**Keywords:** fault diagnosis, bearing, feature extraction, multipoint optimal minimum entropy deconvolution adjusted (MOMEDA)

## Abstract

Due to the early formation of rolling bearing fault characteristics in an environment with strong background noise, the single use of the time-varying filtering empirical mode decomposition (TVFEMD) method is not effective for the extraction of fault characteristics. To solve this problem, a new method for early fault detection of rolling bearings is proposed, which combines multipoint optimal minimum entropy deconvolution adjusted (MOMEDA) with parameter optimization and TVFEMD. Firstly, a new weighted envelope spectrum kurtosis index is constructed using the correlation coefficient and envelope spectrum kurtosis, which is used to identify the effective component and noise component of the bearing fault signal decomposed by TVFEMD, and the intrinsic mode function (IMF) containing rich fault information is selected for reconstruction. Then, a new synthetic impact index (SII) is constructed by combining the maximum value of the autocorrelation function and the kurtosis of the envelope spectrum. The SII index is used as the fitness function of the gray wolf optimization algorithm to optimize the fault period, T, and the filter length, L, of MOMDEA. The signal reconstructed by TVF-EMD undergoes adaptive filtering using the MOMEDA method after parameter optimization. Finally, an envelope spectrum analysis is performed on the signal filtered by the adaptive MOMEDA method to extract fault feature information. The experimental results of the simulated and measured signals indicate that this method can effectively extract early fault features of rolling bearings and has good reliability. Compared to the classical FSK, MCKD, and TVFEMD-MOMEDA methods, the first-order correlated kurtosis (FCK) and fault feature coefficient (FFC) of the filtered signal obtained using the proposed method are the largest, while the sample entropy (SE) and envelope spectrum entropy (ESE) are the smallest.

## 1. Introduction

Rolling bearings, as the most basic parts of rotating machinery, are often prone to wear, cracks, rolling body deformation, and other failures. Therefore, how to discover faults in time and determine the running condition of equipment through the analysis of bearings is of great significance to ensure the safety of operators and avoid significant economic losses of enterprises [1]. Since bearing operation sites usually have strong background interference noise, finding effective methods for extracting faint fault features is the focus of current research in this field.

At present, the method of bearing health detection using time-domain statistical characteristics has been widely studied. Igba et al. [2] used the root mean square value of vibration signals to detect faults, such as bearing pitting and scratches, but they were unable to accurately respond to early bearing faults. Liang et al. [3] utilized the sensitivity of kurtosis to detect early-stage faults and used the kurtosis index of vibration signals to identify faults in the initial stage of bearing failure. To capture the signal characteristics of different stages of faults and to obtain more reliable results by combining different statistical features [4,5], Laissaoui et al. [6] used a combination of root mean square values and kurtosis to track the trend of bearing degradation evolution. Sassi et al. [7] constructed a new index of bearing fault detection based on the empirical relationship between root mean square, kurtosis, peak factor, and peak value. Li et al. [8] used the peak, skewness, kurtosis, and waveform factors of bearing vibration signals to accurately diagnose the types of bearing faults. Liu et al. [9] identified the damage location based on the frequency spectrum of amplitude ratios of bearing fault signals and estimated the bearing damage degree based on effective features in the time-domain vibration signal statistical characteristics. This method could accurately identify the bearing damage location and damage degree at the same time.

The methods of time–frequency analysis have also been widely used in bearing early fault diagnosis, such as the Fourier transform and wavelet transform. However, it is found that there are some shortcomings of the Fourier transform and the wavelet transform, such as spectral leakage, difficulty in choosing the wavelet basis function, and a lack of adaptivity of the noise reduction threshold [10]. Empirical mode decomposition (EMD) adaptively decomposes a signal into a number of intrinsic mode functions with different frequencies and a residual component, with each IMF containing local features of the original signal at different time scales [11]. EMD is a great breakthrough from traditional time–frequency analysis, but there are still deficiencies such as modal confusion, endpoint effects, and sensitivity to noise. Many scholars have made continuous improvements to EMD. Hoseinzadeh et al. [12] proposed ensemble empirical mode decomposition (EEMD), which can reduce modal aliasing of EMD. But there is still some residual noise amplitude in the obtained IMF components, which leads to the generation of false modal components. Chen et al. [13] proposed complementary ensemble empirical mode decomposition (CEEMD). The CEEMD algorithm adds positive and negative paired auxiliary white noises to the original signal, and the phase cancels out at ensemble averaging, which can effectively overcome the problems of large reconstruction errors and poor decomposition completeness of EEMD. However, in the CEEMD algorithm, there are differences in the number of IMFs generated during each EMD decomposition, which leads to difficulties in IMF component alignment when the final set is averaged, resulting in errors. Cai et al. [14] proposed an improved complete ensemble empirical mode decomposition with adaptive noise (CEEMDAN). After resolving an IMF component each time, CEEMDAN adds white noise to the residual value again and completes the decomposition iteratively. CEEMDAN has better mode decomposition results and smaller reconstruction errors. However, the IMFs decomposed by CEEMDAN still contain residual noise, and some “false” modes appear in the early stage of decomposition.

In order to further improve the decomposition performance of the EMD algorithm, Li et al. [15] proposed the time-varying filtering empirical mode decomposition (TVF-EMD) algorithm. Compared to the EMD algorithm, the TVFEMD algorithm adopts the instantaneous amplitude and frequency information, adaptively designs the local cutoff frequency, and then uses the nonuniform B-sample approximation as the time-varying filter, which effectively overcomes the modal aliasing problem of the EMD algorithm and improves the system’s frequency separation performance and stability at a low sampling rate. TVFEMD has been widely used in the field of fault diagnosis in recent years due to its high decomposition efficiency and reconstruction accuracy [16,17]. However, in a strong noise environment or complex system, the obtained fault feature information is early and seriously disturbed by noise, and the signal-to-noise ratio is low. If TVFEMD is directly used to decompose signals, the decomposed IMF components will contain a large amount of high-frequency noise, which leads to poor or no extraction of fault features. Therefore, it is necessary to pre-denoise signals with strong noise in order to highlight the original fault impact component.

The MOMEDA method is a signal noise reduction method that uses a non-iterative approach with the goal of finding the best inverse filter with the maximum D-norm [18]. Yong et al. [19] combined MOMEDA with the Teager energy operator for bearing fault diagnosis and achieved good results, but they did not provide a selection scheme for important parameters in MOMEDA method (such as fault period T and filter length L). The fault period T and filter length L determine the effectiveness of signal filtering via the MOMEDA method. Zhijian Wang et al. [20] used multipoint kurtosis spectrum to determine the fault period T, but did not discuss the influence of filter length on signal enhancement by MOMEDA. Zhou et al. [21] used the advance and retreat method to find the filter length L, but did not explain how to optimize the fault period T. Moreover, it is easy to fall into local optimum when the advance and retreat method searches for the optimal parameters of the filter [22].

Based on the shortcomings of existing TVFEMD and MOMEDA algorithms, in this paper, TVFEMD and MOMEDA are combined to construct a new method of early fault feature detection of rolling bearings. Firstly, to distinguish the fault mode components and noise components after TVF-EMD decomposition, a new weighted envelope kurtosis index is constructed by using the correlation coefficient and envelope kurtosis. This new index is used to screen the IMFs decomposed by TVF-EMD, and the effective IMFs with rich bearing fault information are retained and then reconstructed. Secondly, to overcome the influence of improper selection of the fault period and filter length on the filtering effect of MOMEDA, a new synthetic impact index (SII) is constructed by combining the maximum value of the autocorrelation function and the kurtosis of the envelope spectrum. Taking SII as the objective function of the gray wolf optimization algorithm, the period parameter T and the filter length parameter L are optimized using the gray wolf optimization algorithm, and the adaptive MOMEDA algorithm is constructed. Finally, the bearing fault information is extracted by combining TVF-EMD modal component selection and adaptive MOMEDA filtering. The reconstructed fault signal obtained using TVF-EMD is filtered by the adaptive MOMEDA, and the envelope spectrum of the signal after MOMEDA filtering is analyzed to extract the fault characteristic information. The effectiveness of the proposed method is verified by comparing the simulated and measured bearing fault signals.

The remainder of this paper is structured as follows. Section 2introduces the basic theory of TVF-EMD and the constructed method of weighted envelope spectrum kurtosis (WESK) index to identify the effective IMF components. In Section 3, the adaptive MOMEDA is introduced, and the bearing early fault diagnosis method based on TVFEMD and adaptive MOMEDA is proposed. Section 4 presents the experimental results of the proposed method for simulating bearing ball fault signals. Section 5 presents the experimental results obtained using different methods on the measured bearing outer-race and inner-race fault signals. Finally, the conclusions are drawn in Section 6.

## 2. TVFEMD Algorithm

### 2.1. Principle of TVFEMD Algorithm

TVFEMD essentially constitutes a low-pass filter whose cutoff frequency varies with time. For any given multi-component signal x(t), it can be expressed as a two-component signal after simplification [23]:

(1) $$x(t)=A(t)e^{j\varphi (t)}=a_{1}(t)e^{j\varphi_{1}(t)}+a_{2}(t)e^{j\varphi_{2}(t)}$$

Therefore, only the decomposition process of the two-component signal needs to be considered. The basic process of TVFEMD of two-component signals is as follows [23,24]:

Step 1: Perform the Hilbert transform on x(t) to obtain the amplitude A(t) and phase φ(t) of the complex analytic signal:

(2) $$z(t)=x(t)+j\hat{x}(t)=A(t)e^{j\varphi (t)}$$

Step 2: Find the moments $\left\{t_{\min}\right\}$ and $\left\{t_{\max}\right\}$ at which the minima and maxima of A(t) are located and the amplitudes $A\left(\left\{t_{\min}\right\}\right)$ and $A\left(\left\{t_{\max}\right\}\right)$.

Step 3: The extreme point $A\left(\left\{t_{\min}\right\}\right)$ is interpolated to obtain $\beta_{1}(t)$, and the extreme point $A\left(\left\{t_{\max}\right\}\right)$ is interpolated to obtain $\beta_{2}(t)$.

Step 4: Calculate the instantaneous mean $\alpha_{1}(t)$ and the instantaneous envelope $\alpha_{2}(t)$.

Step 5: Calculate ${\varphi^{\prime }}_{1}(t)$ and ${\varphi^{\prime }}_{2}(t)$ by interpolating $\varphi^{\prime }(t_{\min})A^{2}(t_{\min})$, $\varphi^{\prime }(t_{\max})A^{2}(t_{\max})$, and then calculate the local cutoff frequency:

(3) $${\varphi^{\prime }}_{bis}(t)=\frac{{\varphi^{\prime }}_{1}(t)+{\varphi^{\prime }}_{2}(t)}{2}$$

Step 6: To eliminate the modal aliasing caused by noise and other components, the signal is reconstructed using the cutoff frequency ${\varphi^{\prime }}_{bis}(t)$ to obtain a new signal:

(4) $$h(t)=\cos[\int {\varphi^{\prime }}_{bis}(t)dt]$$

Step 7: Taking the poles of h(t) as nodes, divide h(t) into n segments, each of which has a step size of m. n is called the order of the spline function B. The signal is approximated by B-spline interpolation, and the approximation result m(t) representing the local mean function is obtained.

Step 8: Calculate θ(t) according to Equation (5). For a given bandwidth threshold ξ, if θ(t)<ξ, then x(t) is the IMF; otherwise, set x(t)=x(t)−m(t) and repeat steps (1)~(7). Equation (5) is as follows:

(5) $$\theta (t)=\frac{B_{Loughlin}}{\varphi_{avg}(t)}$$

where $B_{Loughlin}$ and $\varphi_{avg}(t)$ can be calculated using the formulas in reference [23].

### 2.2. Selection of Modal Components

After the bearing fault signal is decomposed by TVFEMD, the number of IMF components obtained is large. Due to the influence of iterative error accumulation, noise, and other factors, only a few IMF components generally contain fault information, and these components are highly correlated with the original signal. The remaining components are usually false components and noise components, which have a low correlation with the original signal. Therefore, the correlation coefficient is often used to reflect the degree of close correlation between a component signal and the original fault signal. If an IMF contains more fault impact components, a more significant fault characteristic frequency will appear in the corresponding envelope spectrum. The envelope spectrum will also deviate significantly from the normal distribution, which eventually leads to an increase in the envelope spectrum kurtosis (ESK) value [25]. Therefore, the ESK index can be used to measure the richness of fault information contained in IMF components. The more fault shock components in an IMF, the larger its kurtosis value is. However, in the case of strong noise interference, the envelope kurtosis index is sensitive to large-amplitude outliers caused by noise and is easily influenced by these outliers. Thus, it sometimes cannot accurately reflect the overall situation of a multi-component signal.

Based on this, the weighted envelope spectrum kurtosis (WESK) index is constructed to identify the effective component and the noise component, where the weight is the correlation coefficient between the IMF component signal and the original signal. The mathematical formulas to obtain the weighted envelope spectrum kurtosis are as follows:

(6) $$ESK=\frac{E[{\left(env\_y-u_{env\_y}\right)}^{4}]}{{\left(E[{\left(env\_y-u_{env\_y}\right)}^{2}]\right)}^{2}}$$

(7) $$\rho (x,y)=\frac{E[(x-u_{x})(y-u_{y})]}{\sigma_{x}\sigma_{y}}$$

(8) WESK=ρ(x,y)⋅ESK

In the formulas, x represents the original signal; y represents the IMF component signal; ESK is the kurtosis of the envelope spectrum; ρ(x,y) is the correlation coefficient; E[⋅] is the mathematical expectation; $\mu_{x}$ and $\mu_{y}$ are the mean values of the original signals and the component signals; $\sigma_{x}$ and $\sigma_{y}$ are the standard deviations of the original signals and the component signals; env_y represents the envelope spectrum of the component signals; and $u_{env\_y}$ represents the mean value of the envelope spectrum of the component signals.

## 3. Adaptive MOMEDA

### 3.1. Principle of MOMEDA Method

Let the signal collected by a vibration sensor be

(9) x=Hy+e

where y is the fault pulse sequence, H is the transfer function, x is the actual collected signal, and e is the noise mixed in the collection process.

The essence of the MOMEDA algorithm is to find the best filter to remove noise from a signal in order to restore the original signal. Based on the D-norm, the MOMEDA algorithm proposes the multipoint D-norm, i.e.,

(10) $$D(y,t)=\frac{1}{\left\|t\right\|}\frac{t^{T}y}{\left\|y\right\|}$$

where t is the target vector to be solved and represents the position and weight of the target pulse. The MOMEDA method is to solve the problem of multipoint D-norm maximization, namely, to solve

(11) $$\underset{f}{\max}D(y,t)=\underset{f}{\max}\frac{t^{T}y}{\left\|y\right\|}$$

Solving the problem presented in Equation (11) is equivalent to solving Equation (12):

(12) $$\frac{d}{df}\left(\frac{t^{T}y}{\left\|y\right\|}\right)=0$$

In the formula, $f=(f_{1},f_{2},\cdots ,f_{L})$, $t=(t_{1},t_{2},\cdots ,t_{N-L})$. Through calculation, it can be inferred that

(13) $$f={\left(X_{0}X_{0}^{T}\right)}^{-1}X_{0}t$$

The final output for the fault impact signal can be expressed as

(14) $$y=X_{0}^{T}f=X_{0}^{T}{\left(X_{0}X_{0}^{T}\right)}^{-1}X_{0}t$$

### 3.2. Analysis of MOMEDA Input Parameters

The input parameters of the MOMEDA algorithm include the window function w, the filter length L, and the search interval t of the fault period $\left[T_{s},T_{f}\right]$. The exact selection of input parameters is essential to improve the performance of MOMEDA.

- The window function w. The window function is used to further extend the target vector, which can improve the clarity of the spectrum and the accuracy of fault shock sequence extraction. In consideration of computational efficiency and the convolution enhancement effect, rectangular windows with a length of 3 are adopted, that is, w=[1,1,1].
- The filter length L. It directly affects the effect of pulse sequence extraction. In order to ensure that the extracted shock sequence can cover the entire frequency band of the fault, the filter length L should meet the following condition [25,26]:
  (15) $$L>2\frac{f_{s}}{f_{*}}$$
  where $f_{s}$ is the sampling frequency of the signal, and $f_{*}$ is the fault characteristic frequency. In this paper, $f_{*}$ represents three kinds of fault characteristic frequency, that is $f_{*}\in \{f_{i},f_{o},f_{b}\}$, where $f_{i}$ is the inner ring fault frequency, $f_{o}$ is the outer ring fault frequency, and $f_{b}$ is the ball fault frequency. However, increasing the filter length will reduce the deconvolution signal length to (N−L+1), which will cause serious loss of fault information. At the same time, increasing L will consume more computing time. Therefore, the selection range of filter length is set as 100≤L≤2000 in this paper.
- Fault cycle search range $\left[T_{s},T_{f}\right]$. $T_{s}$ and $T_{f}$ are the initial and final values of periodic fault search, respectively. According to the calculation formula of bearing characteristic frequency, the characteristic frequency and failure period of each unit (inner ring $f_{i}$, outer ring $f_{o}$ and rolling body $f_{b}$) can be calculated as follows: ($T_{i}=f_{s}/f_{i}$ is the inner ring failure cycle; $T_{o}=f_{s}/f_{o}$ is the failure period of the outer ring; and $T_{b}=f_{s}/f_{b}$ is the failure period of the rolling body. Since the characteristic frequency between each unit of the bearing satisfies $f_{i}>f_{o}>f_{b}$, the failure period satisfies $T_{i}<T_{o}<T_{b}$. When the search interval contains the fault period, the final value $T_{f}$ will not affect the extraction of the fault impact sequence [21]. Therefore, $T_{f}=1000$ is chosen according to the actual operation of bearings. In the experiment, according to the fault forms of different bearing units, the initial value $T_{s}$ of the failure period is selected in the interval range $T_{i}\leq T_{o}\leq T_{b}$.
- Optimize parameters L and $T_{s}$. To select the optimal parameter combination $\left[L,T_{s}\right]$, a new index of multi-objective optimization is constructed to adaptively determine the filter length and the initial value of the fault cycle search. In the time domain, the root mean square of the maximum of autocorrelation function (MOAF) is used to measure the periodic fault impact components contained in the deconvolution signals [26,27]. The core of this index is the autocorrelation function. If the main component of a deconvolved signal is noise, its autocorrelation function will soon decay to 0, and the MOAF value will be very small and close to 0. If the deconvolved signal contains obvious periodic fault impact components, its autocorrelation function is periodic, and the MOAF value is larger. Therefore, the MOFM index is used to measure the extraction effect of deconvolution operation on fault shock sequences, and its expression is as follows:
  (16) $$MOAF=\underset{\tau =1,\cdots ,N}{\max}\{\frac{R^{2}(\tau )}{\tau }\}$$
  where $R(\tau )=\int_{-\infty }^{\infty }x(t)x(t-\tau )dt$, and τ is the delay in the autocorrelation function.

If a deconvolution signal contains more fault impact components, a more significant fault characteristic frequency will appear in the corresponding envelope spectrum. The envelope spectrum will deviate significantly from the normal distribution, which eventually leads to an increase in the envelope spectrum kurtosis (ESK) value. Therefore, ESK, obtained using Equation (6), can be used to measure the richness of fault information contained in the frequency domain of the deconvolved signal.

A new syncretic impact index (SII) is constructed by combining the MOAF and ESK of the deconvolved signal, and it is used as the objective function of parameter optimization in MOMEDA. SII is defined as follows:

(17) SII=MOAF⋅ESK

The maximum value of SII is selected by using the gray wolf optimizer (GWO) [28] to adaptively determine the optimal input parameter combination $\left[T_{s},L\right]$ in MOMEDA so as to improve the deconvolution capability of MOMEDA to the impact of early bearing fault shocks.

### 3.3. MOMEDA with Adaptive Parameters

Based on the above discussion, a new fusion index SII is first constructed as the objective function of parameter optimization in MOMEDA. The optimal parameter $\left[T_{s},L\right]$ of MOMEDA is selected automatically by using GWO. The concrete implementation steps of parameter adaptive MOMEDA algorithm in bearing early fault diagnosis are as follows:

Step 1: TVFEMD is used to decompose the bearing fault vibration signal. The IMF components containing more fault information are selected based on weighted envelope spectral kurtosis (WESK), and the selected IMF components are accumulated as the initial signals.

Step 2: Calculate the failure cycle of each element of the bearing. According to the principle of parameter selection in Section 3.2, the search range $T_{i}<T_{o}<T_{b}$ and the final value $T_{f}=1000$ of the initial value of the fault cycle are set, the range of the filter length is taken as 100≤L≤2000, and the rectangular window with length w=3 is selected as the window function.

Step 3: Set the population size and the maximum number of iterations, and initialize the wolf pack position. The population size of the gray wolf optimization algorithm is generally between 10 and 50. In addition, if the maximum number of iterations is set too small, it is easy to fall into the local optimal solution. If the setting is too large, the running time of the algorithm will increase, and its efficiency will be reduced. In this paper, we set the wolf population size as 20 and the maximum number of iterations as 20, and we randomly generate 20 positions as the initial positions of the wolves in the range of the search for superiority.

Step 4: The fitness function value of each wolf at its current position is calculated according to Equation (17), and the top 3 wolves with the best fitness value are saved as wolves α,β,δ.

Step 5: Update the gray wolf position according to Equations (18) and (19) as follows:

(18) $$\left\{\begin{array}{c} X_{1}=X_{\alpha }-A_{1}\cdot D_{\alpha }\\ X_{2}=X_{\beta }-A_{2}\cdot D_{\beta }\\ X_{3}=X_{\delta }-A_{3}\cdot D_{\delta } \end{array}\right.$$

(19) $$X(t+1)=\frac{X_{1}+X_{2}+X_{3}}{3}$$

where A is the cooperativity coefficient vector; $D_{\alpha }$, $D_{\beta }$, $D_{\delta }$ are the distances between wolves α,β,δ and food, respectively; and $X_{\alpha }$, $X_{\beta }$, $X_{\delta }$ are the position vectors of wolves α,β,δ, respectively.

Step 6: Calculate the fitness value of all updated gray wolves and compare it with the current fitness function value of wolves α,β,δ. If the result is better, update wolves α,β,δ.

Step 7: Repeat Step 4 to Step 6 until the iteration is terminated, and the output is the best parameter $\left[T_{s}^{*},L^{*}\right]$.

Step 8: The fault signals processed by TVFEMD are deconvolved by using MOMEDA with the optimized parameters.

Step 9: The envelope demodulation of the deconvolution signal is carried out. The fault characteristic frequencies are extracted and compared with the theoretical values to distinguish the fault type of the bearing.

**Figure 1:**
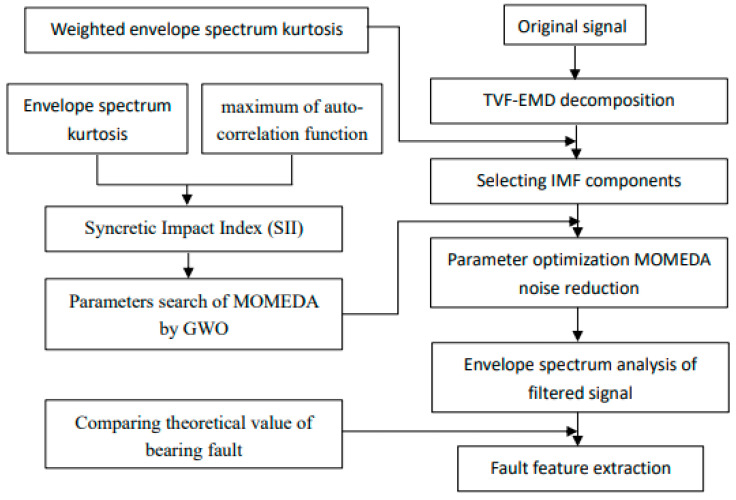
Flowchart of fault diagnosis based on TVFEMD and adaptive MOMEDA.

The flow of the bearing early fault diagnosis method based on TVFEMD and parameter adaptive MOMEDA is shown in Figure 1.

## 4. Simulation Signal Analysis

**Figure 2:**
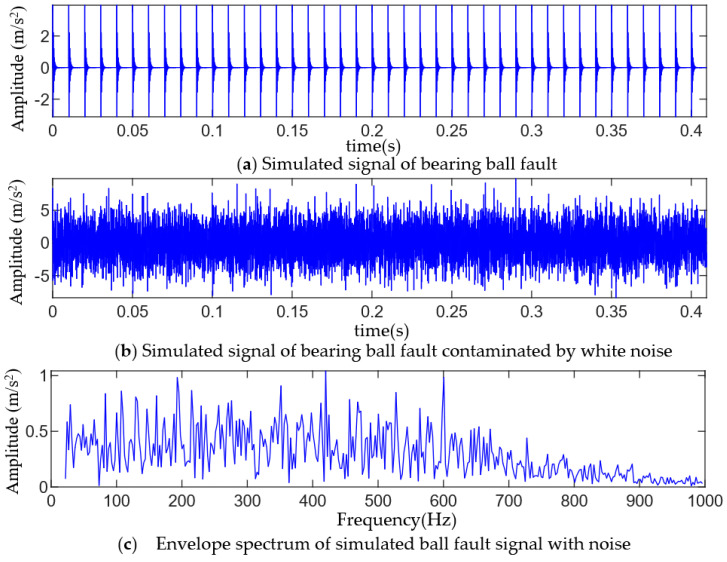
Time-domain waveform and envelope spectrum of a simulated fault signal.

To verify the effectiveness of the proposed method, a bearing ball fault periodic pulse signal under strong background noise was constructed, and the simulation experiment was carried out. The simulation model is as follows:

(20) $$\left\{\begin{array}{c} x(t)=y_{0}\exp(-2\pi \varepsilon f_{n}t)\sin(2\pi f_{n}\sqrt{1-\varepsilon^{2}}t)\\ y(t)=x(t)+n(t) \end{array}\right.$$

where the inherent frequency of the system is $f_{n}=3000$, the damping coefficient is ε=0.25, the displacement constant is $y_{0}=4$, and the repetition period of fault impact is T=0.01 s, namely, the fault frequency is $f_{b}=100$ Hz, and the sampling frequency is $f_{s}=20$ kHz. The number of sampling points N=8192, and t is the sampling time, while n(t) is the white noise signal. To simulate strong background noise, the signal-to-noise ratio of the whole signal after adding white noise is −14 dB. The time domain waveform and envelope spectrum of the simulated signal are shown in Figure 2. It can be seen from Figure 2b that the fault pulse sequence in the signal is almost all submerged by noise, and there is no significant prominent frequency spectrum in the envelope spectrum of Figure 2c, and thus, the fault characteristic information cannot be identified.

**Figure 3:**
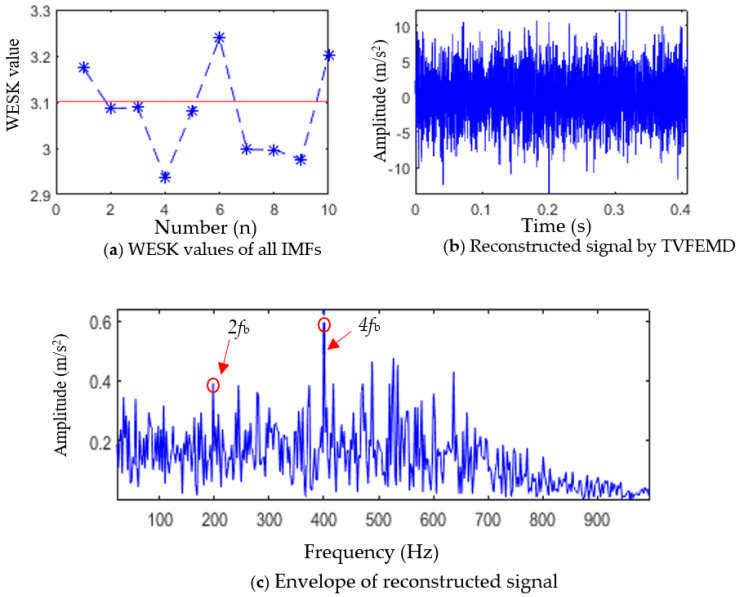
Selection of IMF components and reconstruction of selected IMFs.

First, TVFEMD was used to decompose the simulated signal containing noise, and the weighted envelope spectrum kurtosis (WESK) of each IMF was calculated (as shown in Figure 3a). Then, IMF components whose WESK value is greater than the average of all IMFs were selected for reconstruction, that is, IMF1, IMF6, and IMF10. The reconstructed signal is shown in Figure 3b, and an envelope spectrum analysis of the reconstructed signal was performed, the result of which is shown in Figure 3c.

As shown in Figure 3b,c, TVFEMD reconstruction improves the envelope spectrum characteristics of the fault signal to a certain extent, and a part of the fault characteristic frequencies can be extracted. However, in the envelope spectrum of the TVFEMD-reconstructed signal, only $2f_{b}$ and $4f_{b}$ can be extracted, and the amplitude of interference frequency in the envelope spectrum is also larger. Generally speaking, a satisfactory fault characteristic frequency extraction effect is not obtained.

At this stage, the proposed adaptive MOMEDA method was used to further process the reconstructed signal in Figure 3b.

**Figure 4:**
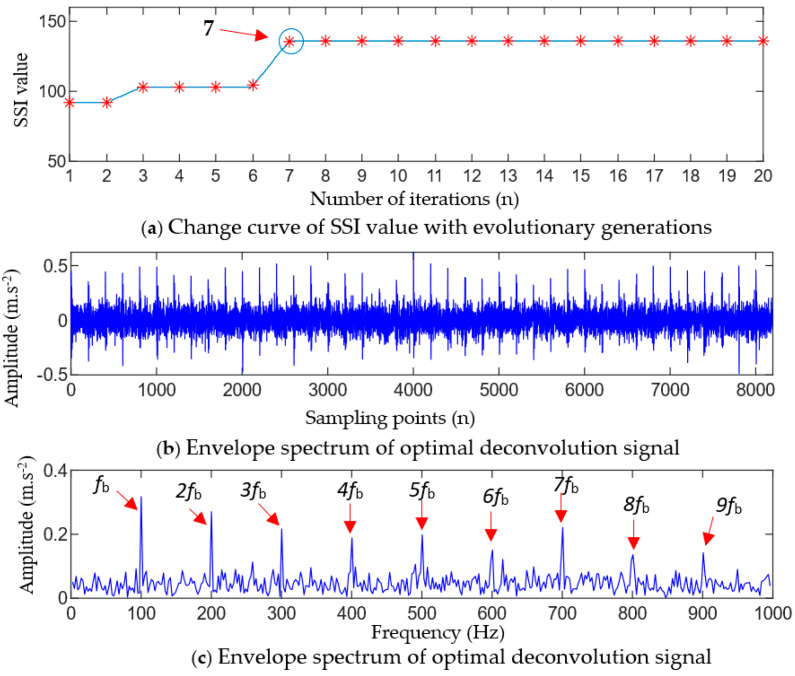
The optimal deconvolution results obtained using the GWO-MOMEDA method.

Firstly, according to the principle of parameter selection in Section 3.2, the value ranges of the fault period $T_{s}$ and filtering length L were set as $50\leq T_{s}\leq 200$ Hz and 100≤L≤2000, respectively. The GWO algorithm was used to solve the optimal parameter combination $\left[T_{s}^{*},L^{*}\right]$ of MOMEDA. Figure 4a shows the relationship between the SII value of the deconvolution signal and the evolution algebra of the population. It can be seen from Figure 4a that the maximum SII value of the deconvolution signal is obtained when the population has evolved to the seventh generation during the GWO optimization process, and the optimal parameter combination $\left[T_{s},L\right]$ searched is [100, 1375]. Then, the MOMEDA with optimal parameters was used to denoise the signal in Figure 3b, and an envelope spectrum analysis was performed on the denoised signal. The MOMEDA-denoised signal and its envelope spectrum are shown in Figure 4b,c.

As can be seen from Figure 4b,c, the waveform of the signal after adaptive MOMEDA filtering clearly appears to have the periodic impact feature of an equal interval. In the corresponding envelope spectrum, there are more prominent spectral peaks at the characteristic frequency $f_{b}$ and the multiple frequency $f_{b}(n=2,3,\cdots ,9)$ of the ball fault. It can be judged that the bearing ball has a fault, which is consistent with the simulation results.

**Figure 5:**
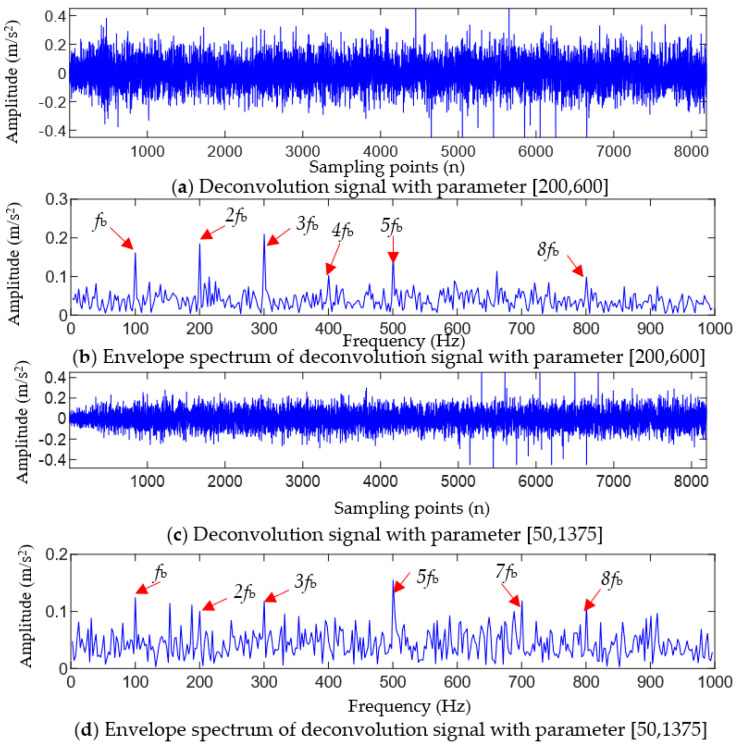
Analysis results of a simulated signal after changing the parameters of MOMEDA.

To verify the accuracy of the optimal parameter combination obtained using the method proposed in this article, a certain parameter in A was randomly replaced, and the TVFEMD-reconstructed signal in Figure 3b was processed using MOMEDA after replacing the parameter. Figure 5a,b show the results obtained by changing the filter length L in the optimal parameter combination [200, 1375] to 600. Compared to Figure 4b, the results of MOMEDA filtering with L = 600 show a significant increase in noise residue and a certain degree of reduction in impact characteristics. In the envelope spectrum corresponding to Figure 5b, only the spectral lines at $f_{b}\sim 5f_{b}$ and $8f_{b}$ are relatively obvious, but the amplitudes of the spectral lines are lower than those in Figure 4c.

Figure 5c,d show the results of changing the fault period $T_{s}$ in the optimal parameter combination [200, 1375] to 50 Hz, and it can be seen that the filtering effect of MOMEDA is also affected when $T_{s}$ is changed to 50. When $T_{s}=50$ Hz, it can be seen from Figure 5c that noise significantly increases after MOMEDA filtering. From Figure 5d, it can be seen that the envelope spectrum amplitude at the integer multiples of the ball fault frequency significantly decreases, and only the spectral lines at $f_{b}\sim 3f_{b}$, $5f_{b}$ and $7f_{b}\sim 8f_{b}$ can be observed, but the amplitudes of the spectral lines are lower than those in Figure 4c. The above results show that the random selection of parameters has a serious impact on the deconvolution effect, resulting in a target frequency that cannot be clearly and completely extracted. The proposed TVFEMD-GWO method can effectively solve the problem of parameter selection and achieve an optimal deconvolution of fault shock.

**Figure 6:**
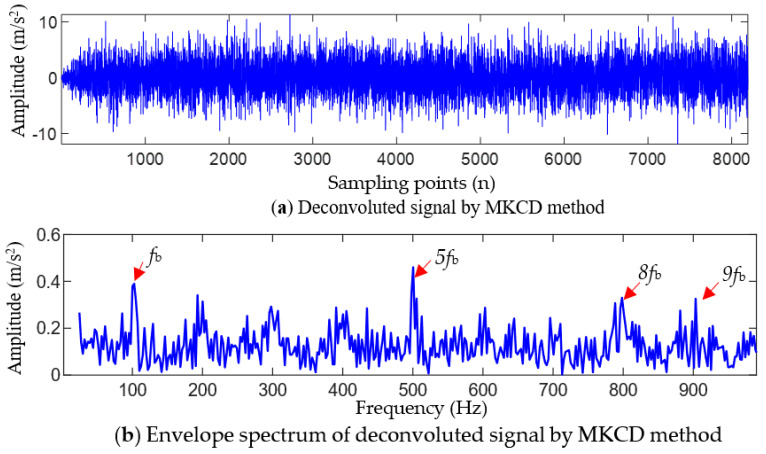
Analysis results of the simulated signal using the MKCD method.

**Figure 7:**
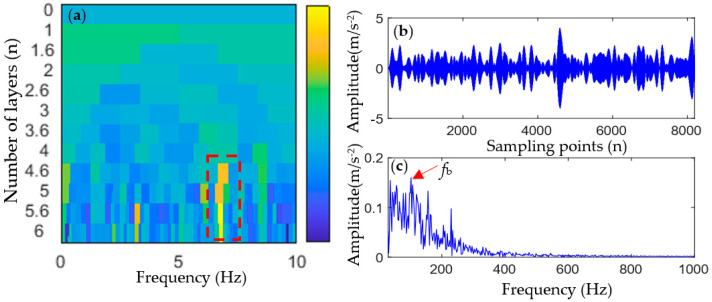
Analysis results of the simulated signal using the FSK method: (**a**) fast spectral kurtosis graph; (**b**) filtered signal after using FSK; and (**c**) envelope spectrum of the filtered signal after using FSK.

For comparison, the maximum correlation kurtosis deconvolution (MCKD) method [29] and the fast spectral kurtosis (FSK) graph method [30] were used to process the simulated signals with noise. Figure 6 shows the processing result of the MCKD method. It can be seen from Figure 6a,b that the signal waveform after deconvolution using MCKD presents early impact features, but the regularity of these impacts is not significant. Only the ball fault frequency $f_{b}$, the 5-order harmonic ($5f_{b}$), the 8-order harmonic ($8f_{b}$), and the 9-order harmonic ($9f_{b}$) of the ball fault can be seen in the envelope spectrum, but only the 1-order, 5-order, and 9-order harmonics are relatively clear. Figure 7 shows the filtered results of the IFSK method. From Figure 7, it can be seen that there are no obvious periodic impact features in the waveform of the filtered signal, and only 1-order harmonic ($f_{b}$) of the ball fault feature frequency can be extracted from the envelope spectrum. Comparing the analysis results of TVFEMD-GWO-MOMEDA in Figure 4, it can be seen that both the MCKD and IFSK methods are difficult to extract obvious fault feature information of the bearing ball, and the processing results are not ideal.

To evaluate and compare the effectiveness of the proposed method, the first-order correlation kurtosis (FCK) [30], fault feature coefficient (FFC) [31], sample entropy (SE) [32], and envelope spectrum entropy (ESE) [33] were introduced into the quantitative analysis of the proposed method. The FCK [30] was used to evaluate the enhancement effect of periodic shock features in signals, and its expression is as follows:

(21) $$FCK=\sum_{n=1}^{N}{\left(x_{n}x_{n-T}\right)}^{2}/\sum_{n=1}^{N}{\left(x_{n}^{2}\right)}^{2}$$

where $x_{n}$ is the analyzed signal and T is the failure period. The FFC [31] was used to evaluate the performance of different methods in the frequency domain. FCC is defined as the ratio of the amplitude of the fault characteristic frequency of the envelope spectrum to the sum of the amplitude of the envelope spectrum, which can accurately evaluate the fault frequency of the envelope spectrum and the significance of the frequency multiplications. The calculation formula for FFC [34] is as follows:

$$FFC=\frac{S(f)+S(2f)+\cdots +S(5f)}{\sum S}$$

where f is the characteristic frequency of bearing failure, S is the amplitude of the envelope spectrum of the analyzed signal, and S(if) is

(22) S(if)=max[S(if−0.02f,if+0.02f)], i=1,2,⋯,K

FCK can effectively evaluate the enhancement effect of periodic impact features on filtered signals. A larger FCK indicates a stronger fault periodic impact feature and, thus, a better filtering effect. FFC can accurately evaluate the significance of fault feature frequency and its multiplier in the envelope spectrum. A larger FFC indicates that the fault feature frequency and its multiplier in the filtered signal’s envelope spectrum are more significant. Therefore, a larger FFC value is better.

SE can reflect the complexity of a filtered signal, and the larger the SE value, the higher the randomness of the signal and the more noise components it contains; the smaller the SE value, the stronger the regularity of the filtered fault signal and the fewer noise components. ESE can effectively characterize the uniformity of periodic pulses in a fault signal. When more uniform pulses are detected in the filtered signal, the envelope spectrum is cleaner, and the entropy of the envelope spectrum is smaller. Therefore, a smaller ESE value for the filtered signal is better.

For the simulated fault signals, the quantitative evaluation index values of the five methods are shown in Table 1. According to the comparison results of the quantitative indicators in Table 1, it can be seen that the FCK and FFC values of the filtered signal using the proposed method are the largest, while the SE and ESE values are the smallest. Compared to the MCKD, FSK, TVFEMD-MOMEDA (L = 600), and TVFEMD-MOMEDA (Ts = 50) methods, the FCK value of the proposed method increased by 493.55%, 388.50%, 76.36%, and 155.56%, respectively; the FFC value increased by 463.30%, 332.39%, 62.86%, and 108.13%, respectively; the SE value decreased by 24.21%, 19.19%, 11.84%, and 8.39%, respectively; and the ESE value decreased by 24.26%, 23.44%, 10.95%, and 14.20%, respectively. These results indicate that combining TVFEMD and adaptive MOMEDA can effectively filter the interference noise after filtering the measured fault signal, enhance the periodic impact characteristics caused by the fault in the filtered signal, and effectively improve the significance of the fault characteristic frequency in the filtered signal envelope spectrum.

## 5. Measured Signal Analysis

### 5.1. Introduction of the Experimental Platform

**Figure 8:**
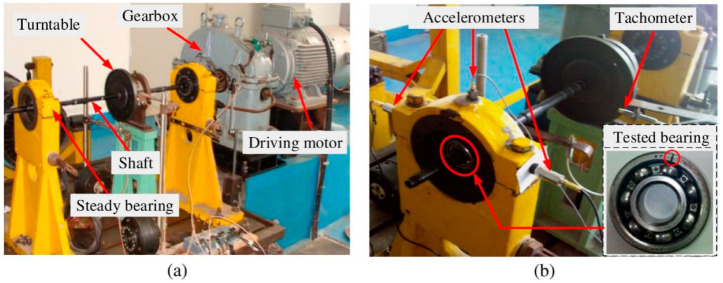
(**a**) The test rig of the roller bearing, and (**b**) sensor locations and the tested bearing with an outer-race defect.

The experimental data were obtained using the MFS mechanical failure comprehensive simulation test bench manufactured by SpectraQuest [35], Richmond, VA, USA, as shown in Figure 8. The test bench is mainly composed of an experimental bench base, a controller, a motor, a bearing base, and different types of rolling bearings, couplings, and rotating shafts. The specific parameters of the tested bearing are shown in Table 2.

The test bench can simulate the failure of the bearing outer ring, inner ring, and rolling body, and the bearing vibration signal can be obtained via the acceleration sensor. When collecting vibration signals of rolling bearings, the sampling frequency is set at 25,600 Hz, and the characteristic frequency of various fault types can be calculated using Equation (23) according to the bearing parameters:

(23) $$\left\{\begin{array}{c} f_{i}=\frac{z}{2}(1+\frac{d}{D}\cos\alpha )f_{r}\\ f_{o}=\frac{z}{2}(1-\frac{d}{D}\cos\alpha )f_{r}\\ f_{b}=\frac{D}{2d}[1-{\left(\frac{d}{D}\right)}^{2}\cos^{2}\alpha ]f_{r} \end{array}\right.$$

where $f_{i}$, $f_{o}$, $f_{b}$ are the inner ring fault characteristic frequency of the rolling bearing, the outer ring fault characteristic frequency, and the ball fault characteristic frequency, respectively; Z is the number of balls; d is the diameter of the rolling bearing; D is the pitch diameter; $f_{r}$ represents the shaft frequency of the bearing; and α represents the contact angle.

During the experiment, the bearing fault was simulated by using electric discharge machining to simulate a single-point damage fault as follows: (1) To simulate the outer ring fault of the bearing, the bearing inner ring and the ball were kept intact before the experiment, and the outer ring was artificially processed with a slight dent (damage depth of 0.59 mm and damage width of 0.11 mm) as the early fault of the bearing outer race. (2). To simulate the inner ring fault of the bearing, the bearing outer ring and the ball were kept intact before the experiment, and the inner ring was artificially processed with a slight dent (damage depth of 1.05 mm and damage width of 0.17 mm) as the early inner-race fault of the bearing.

**Figure 9:**
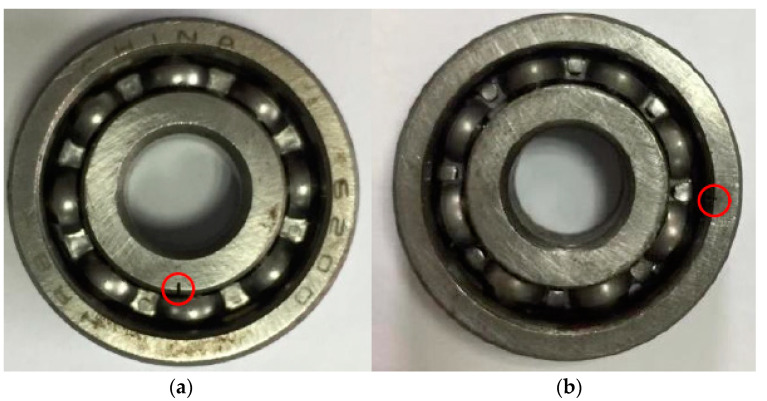
Bearing inner-race and outer-race faults. (**a**) Inner-race fault; (**b**) outer-race fault.

Figure 9a,b show the images of the actual damage of the bearing inner-race fault and outer-race fault. The theoretical calculation shows that the characteristic frequency of the outer ring fault is 81.125 Hz, the characteristic frequency of the inner ring fault is 118.875 Hz, and the shaft frequency of the bearing is 25 Hz.

### 5.2. Analysis of the Bearing Outer Ring Fault Signal

**Figure 10:**
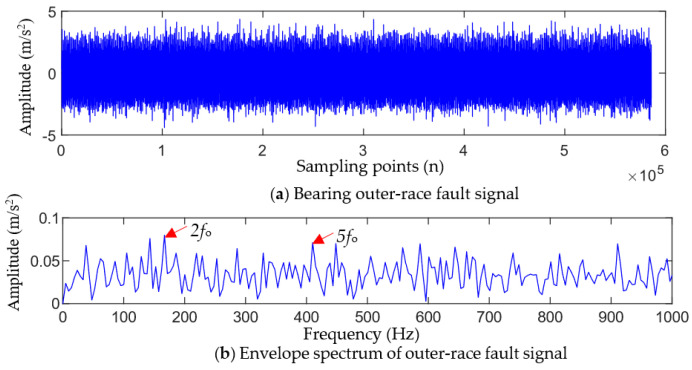
Bearing outer-race fault signal and its envelope spectrum.

Figure 10 shows the bearing outer ring fault signal collected by the vertical sensor and its envelope spectrum. As can be seen from Figure 10, it is difficult to observe regular impact characteristics in the time-domain waveform of the outer-race fault. The envelope spectrum also does not show the corresponding characteristic frequency of the outer-race fault (only showing the 2-order harmonic and 5-order harmonic, which are insignificant), and noise and other unrelated interference frequencies are more prominent. Therefore, the characteristic information of the bearing fault cannot be extracted from the original fault signals based on traditional time-domain analysis and envelope spectrum.

To reveal the early fault symptoms caused by the damage of the bearing outer ring hidden in the dynamic signal, the proposed method was used to analyze the bearing outer-race fault signal.

**Figure 11:**
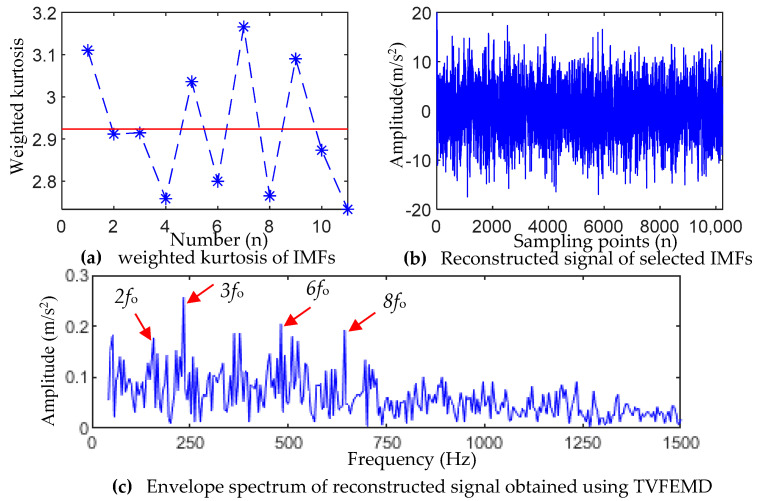
Outer-race fault analysis results of the reconstructed signal using TVFEMD.

TVFEMD was used to decompose the outer-race fault signal and calculate the weighted kurtosis value of each IMF component, and the results are shown in Figure 11a. IMF1, IMF5, IMF8, and IMF9 were selected to reconstruct the signal, and the envelope spectrum of the reconstructed signal was calculated, and the results are shown in Figure 11b,c. It can be seen that the reconstructed TVFEMD signal can remove the interference of noise to a certain extent, and the partial harmonic frequency ($2f_{o}$, $3f_{o}$, $6f_{o}$, and $8f_{o}$) of the outer-race fault can be observed in its envelope spectrum. However, the envelope spectrum of the reconstructed signal can only extract two, three, four, and eight times the fault characteristic frequency, and the amplitude of the interference frequency is also large. Therefore, satisfactory results of fault feature frequency extraction are not obtained.

According to the parameter selection principle in Section 2.2, the initial values of the fault period $T_{s}$ and the filter length L are set to $T_{i}\leq T_{s}\leq T_{b}$ and 100≤L≤2000, respectively. According to the calculation formula of the bearing failure period theory, $168.2\leq T_{s}\leq 312.9$ Hz is obtained. The GWO algorithm was used to optimize the parameters of MOMEDA, and it can be seen from Figure 11a that the maximum SII value of the deconvolution signal is obtained when the population evolves to the 11th generation, while the optimal parameter combination $\left[T_{s}^{*},L^{*}\right]$ searched is [247.27, 1602].

**Figure 12:**
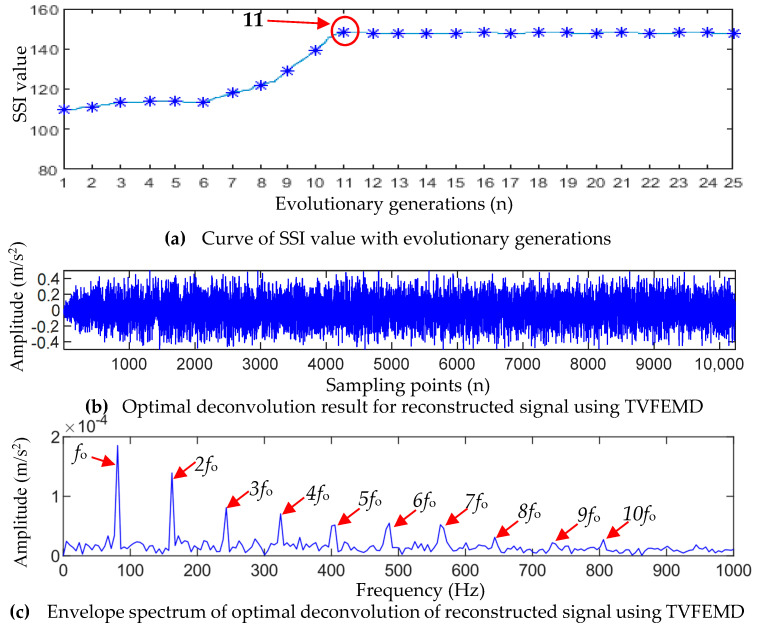
Outer-race fault analysis results of the proposed TVFEMD-GWO-MOMEDA.

The signal reconstructed by using TVFEMD was denoised using optimal-parameter MOMEDA, and the envelope spectrum of the denoised signal was calculated. Figure 12b,c show the denoised signal and its envelope spectrum. It can be seen that after TVFEMD reconstruction and deconvolution operation, the periodic shock feature hidden in the dynamic signal is clearly revealed, and the noise and other interference are effectively removed. In the envelope spectrum, the characteristic frequency ($f_{o}$) of the outer-race fault and its harmonics ($nf_{o},n=2,3,\cdots ,8$) have obvious spectral lines, which are dominant in the whole spectrum. Therefore, the above analysis results show that there is a fault in the bearing outer race, which is consistent with the experimental setting.

To test the reliability of the optimal parameter combination $\left[T_{s}^{*},L^{*}\right]$ in the actual bearing outer-race fault signal, one of the parameters in $\left[T_{s}^{*},L^{*}\right]$ was randomly changed, and the original MOMEDA method, after changing the parameter, was used to process the outer-race fault signal.

**Figure 13:**
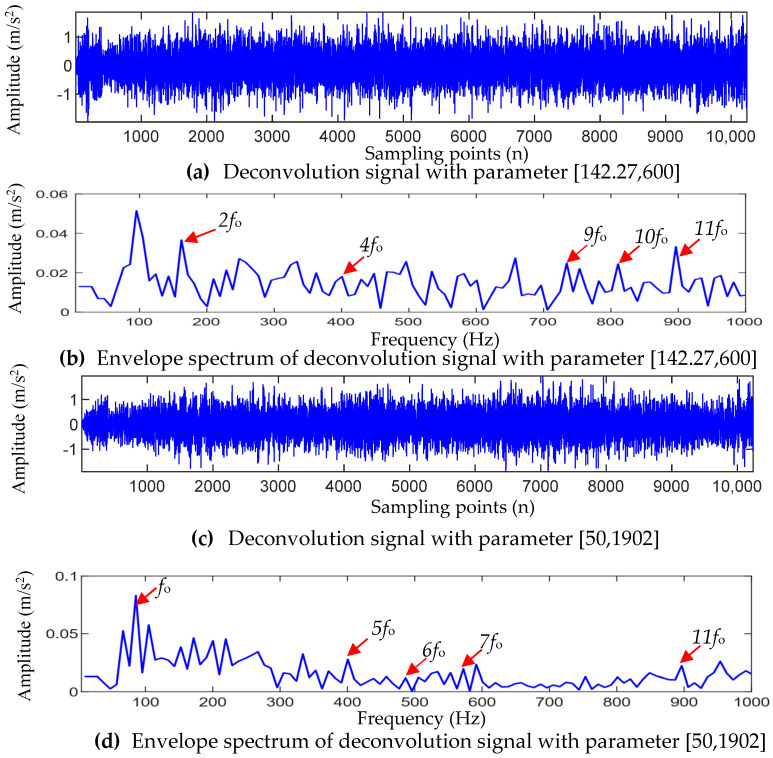
Analysis results of the outer-race fault signal by changing the parameters of MOMEDA.

After the filter length L in the optimal parameter combination [147.27, 1902] was changed to 600, the MOMEDA method was used to filter the TVFEMD-reconstructed signal, and the results are shown in Figure 13a,b. Compared to Figure 12b, the reconstructed signal in Figure 13a clearly contains more noise. Meanwhile, in the envelope spectrum corresponding to Figure 13b, only the spectral lines of $2f_{o}$, $4f_{o}$, $9f_{o}$, $10f_{o}$, and $11f_{o}$ can be observed. Moreover, due to noise interference, the spectral lines of the characteristic frequency $nf_{o}(n=4,9,10)$ are not obvious, and the overall amplitude is lower than that in Figure 12c. After changing the initial value of the fault cycle in the optimal parameter combination [147.27, 1902] to 50 Hz, MOMEDA was used to filter the reconstructed signal of TVFEMD. The results are shown in Figure 13c,d. As can be seen from Figure 13c, the filtered signal based on MOMEDA with the parameter combination [50, 1902] still has relatively serious noise interference. From Figure 13d, it can be seen that only the spectral line of $f_{o}$, $5f_{o}$, $6f_{o}$, $7f_{o}$, and $11f_{o}$ can be observed in the corresponding envelope spectrum, and the amplitude of the spectral lines in $5f_{o}$, $6f_{o}$, and $7f_{o}$ are not significant. It can be seen that after changing the initial value of the optimal fault period $T_{s}$, the original MOMEDA method could not extract the early fault features of the outer-race well.

**Figure 14:**
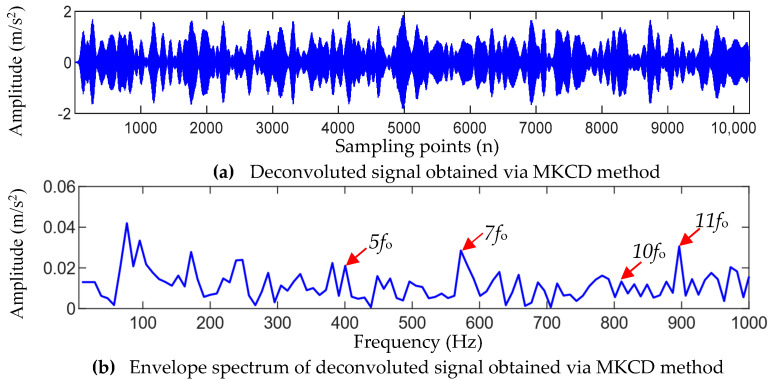
Analysis results of bearing outer-race fault signal using MKCD method.

**Figure 15:**
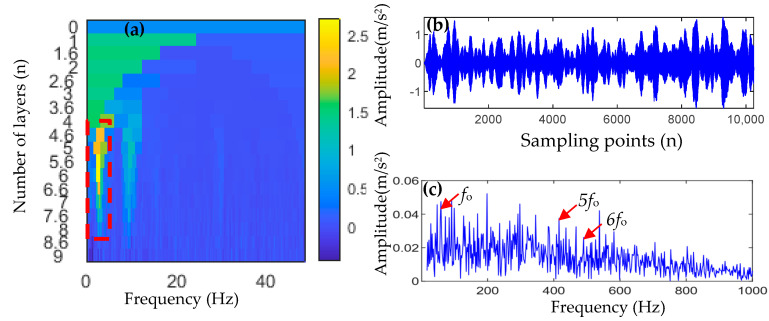
Analysis results of bearing outer-race fault signal using the FSK method: (**a**) fast spectral kurtosis graph, (**b**) filtered signal using FSK, and (**c**) envelope spectrum of a filtered signal.

As a comparison, the MCKD method and the FSK method were used to analyze the fault signals of the bearing outer race. Figure 13 shows the processing results of the MCKD method. It can be seen that MCKD only extracts a part of the impact components, and only the 3-, 7-, 10-, and 11-order harmonic ($3f_{o}$, $7f_{o}$, $10f_{o}$, and $11f_{o}$) of the outer-race fault frequency can be observed in the envelope spectrum shown in Figure 14b. Overall, the outer-race fault frequency $f_{o}$ and its harmonic $nf_{o}$ are not easily recognized. Figure 15 shows the results of filtering using the FSK method. From Figure 15b, it can be seen that after FSK filtering, there are shock features with varying strengths in the signal, but the periodicity of these shocks is not clear. In the envelope spectrum shown in Figure 15c, only the outer-race fault frequency $f_{o}$ and the 5- and 6-order harmonics can be observed. Comparing the analysis results obtained using TVFEMD-GWO-MOMEDA in Figure 12c, it can be seen that both the MCKD method and the FSK method have difficulties extracting the early periodic impact features of the bearing outer-race fault clearly, and the processing results are not as ideal as the TVFEMD-GWO-MOMEDA method.

For the measured outer-race fault signals, the quantitative evaluation index values of the five methods are shown in Table 3. According to the comparison results of the quantitative indicators in Table 3, it can be seen that the FCK and FFC values of the filtered signal obtained using the proposed method are the largest, while the SE and ESE values are the smallest. Compared to the MCKD, FSK, TVFEMD-MOMEDA (L = 600), and TVFEMD-MOMEDA (Ts = 50 Hz) methods, the FCK value of the proposed method increased by 437.35%, 129.90%, 50.17%, and 87.39%, respectively; the FFC value increased by 836.58%, 276.47%, 67.69%, and 100.13%, respectively; the SE value decreased by 23.20%, 21.90%, 12.52%, and 14.39%, respectively; and the ESE value decreased by 19.43%, 17.46%, 10.70%, and 12.71%, respectively. The results indicate that the TVFEMD-GWO-MOMEDA method can effectively filter the interference noise for the measured outer-race fault signal, enhance the periodic impact characteristics caused by a fault in the filtered signal, and effectively improve the significance of the fault characteristic frequency in the envelope spectrum.

### 5.3. Bearing Inner-Race Fault Signal Analysis

**Figure 16:**
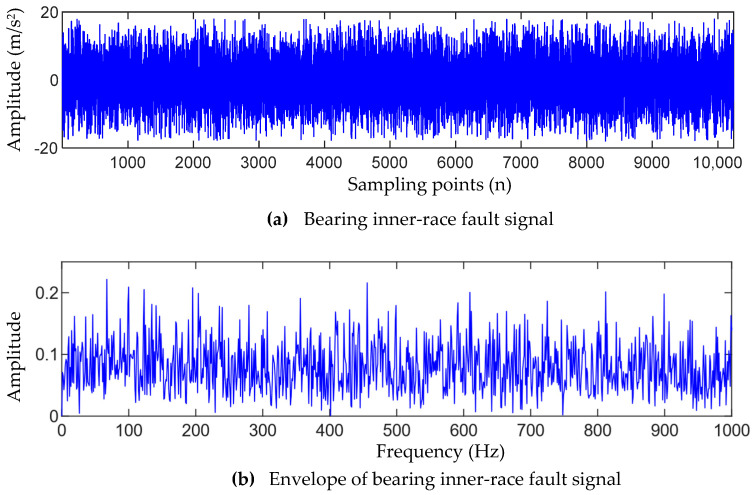
Bearing inner-race fault signal and its envelope spectrum.

Figure 16a shows the fault signal of the bearing inner race detected by the vertical direction sensor, and Figure 16b shows the envelope spectrum of the inner-race fault signal. From Figure 16b, it can be seen that it is difficult to extract the corresponding inner-race fault characteristic information from its envelope spectrum.

**Figure 17:**
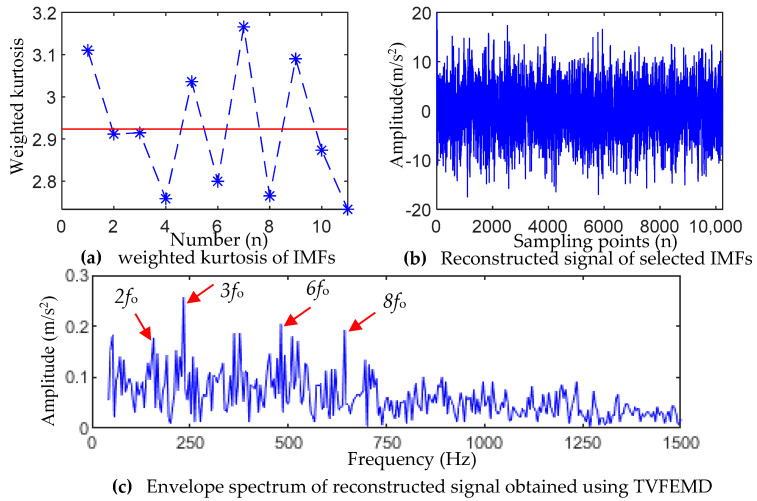
Inner-race fault analysis results of a reconstructed signal using TVFEMD.

TVFEMD was used to decompose the inner-race fault signal and calculate the weighted kurtosis value of each IMF component, and the results are shown in Figure 17a. IMF3, IMF5, IMF6, and IMF7 were selected to reconstruct the signal, and the envelope spectrum of the reconstructed signal was calculated, with the results being shown in Figure 17b,c. It can be seen that the envelope spectrum of the reconstructed signal can only extract the 1-, 2-, 4-, and 5-order harmonics of the inner fault frequency, and the amplitude of the interference frequency is also large. Therefore, satisfactory results of inner fault frequency extraction are not obtained.

**Figure 18:**
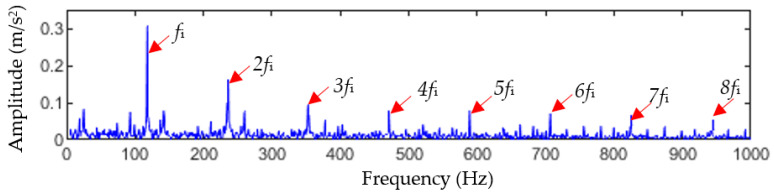
Inner-race fault envelope spectrum of TVFEMD-GWO-MOMEDA.

The GWO-MOMEDA method was used to denoise the reconstructed signal obtained using TVFEMD, and the initial values of the fault period $T_{s}$ and the filter length L were set to a range of $168.2\leq T_{s}\leq 312.9$ Hz and 100≤L≤2000, respectively, while the optimal parameter combination $\left[T_{s}^{*},L^{*}\right]$ searched was [234.3, 1348]. The signal reconstructed based on TVFEMD was denoised by using the optimal-parameter MOMEDA, and the envelope spectrum of the denoised signal was calculated, which is shown in Figure 18. In the envelope spectrum, the inner fault frequency $f_{i}$ and its harmonics ($nf_{i},n=2,3,\cdots ,8$) have obvious spectral lines. Therefore, the above analysis results show that there is a fault in the bearing inner race, which is consistent with the experimental setting.

In order to test the reliability of the optimal parameter combination $\left[T_{s}^{*},L^{*}\right]$ in the actual bearing inner-race fault signal, one of the parameters in $\left[T_{s}^{*},L^{*}\right]$ was randomly changed, and the original MOMEDA method, after changing the parameter, was used to process the inner-race fault signal.

**Figure 19:**
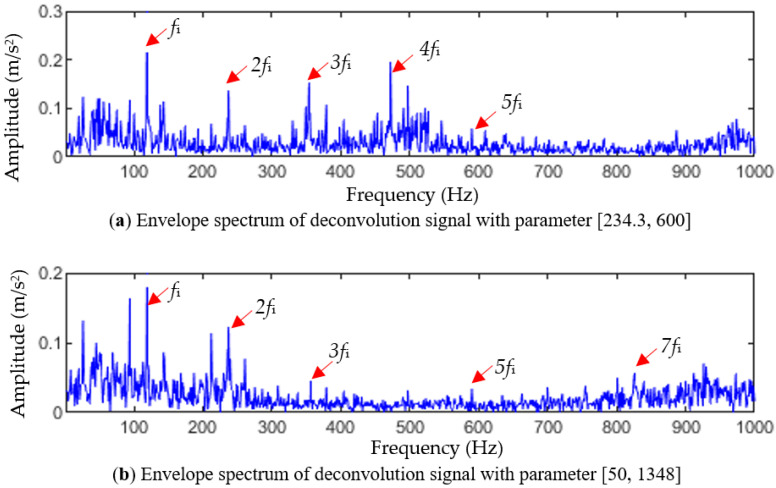
Analysis results of the inner-race fault by changing the parameters of MOMEDA.

Figure 19a shows the envelope spectrum obtained by denoising the TVFEMD reconstruction results after changing the filter length L in the optimal parameter combination [234.31348] to 600. Compared to Figure 18, it can be seen that due to the presence of more noises in the reconstructed signal, the harmonics of the inner-race fault frequency in the envelope spectrum become blurred. Only the inner-race fault frequency $f_{i}$ and the 2-, 3-, 4-, and 5-order harmonics ($2f_{i}$, $3f_{i}$, $4f_{i}$, $5f_{i}$) can be observed, and the overall amplitude of the spectral lines is lower than that in Figure 18.

Figure 19b shows the envelope spectrum of the reconstructed signal after changing the fault period $T_{s}$ in the optimal parameter combination [234.31348] to 50 Hz. From Figure 19b, it can be seen that only the inner-race fault frequency $f_{i}$, and the 2-, 3-, 5-, and 7-order harmonics ($2f_{i}$, $3f_{i}$, $5f_{i}$, $7f_{i}$) can be observed, and the amplitudes of the 3-, 5-, and 7-order harmonics are also small. From the above experimental results, it can be seen that after changing $T_{s}$ or L in the optimal parameter combination, the inner circle fault feature extraction performance of the TVFEMD-MOMEDA method will be greatly affected.

**Figure 20:**
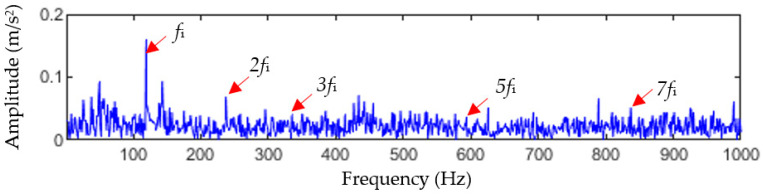
Analysis results of the bearing inner-race fault using the MCKD method.

**Figure 21:**
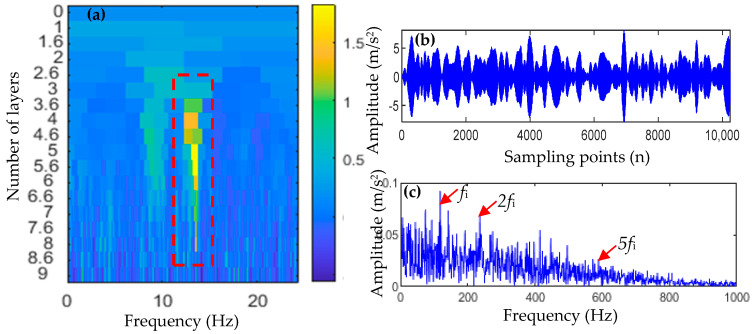
Analysis results of the bearing inner-race fault signal using the FSK method: (**a**) fast spectral kurtosis graph, (**b**) filtered signal using FSK, and (**c**) envelope spectrum of the filtered signal by FSK.

As a comparison, the MCKD method and the FSK method were used to analyze the fault signals of the bearing inner race. Figure 20 shows the processing results of the MCKD method. It can be seen that MCKD only extracts the inner-race fault characteristic frequency $f_{i}$ and the 2-, 3-, 5-, and 7-order harmonics ($2f_{i}$, $3f_{i}$, $5f_{i}$, and $7f_{i}$), but $2f_{i}$, $3f_{i}$, $5f_{i}$, and $7f_{i}$ are not easily recognized. Figure 21 shows the results of filtering using the FSK method. As shown in Figure 21c, only the inner-race fault frequency $f_{i}$ can be observed. Compared to the analysis results of TVFEMD-GWO-MOMEDA in Figure 18, it can be seen that both the MCKD method and the FSK method have difficulties extracting the early periodic impact features of bearing inner-race fault clearly, and the recognition results of the inner-race fault are not as ideal as the TVFEMD-GWO-MOMEDA method.

The evaluation index results of the five filtering methods for the measured inner-race fault signal are shown in Table 4. It can be seen from Table 4 that for the measured bearing inner-race fault signal, the FCK and FFC values of the proposed method are also the largest, while the SE and ESE values are also the smallest. The results indicate that the proposed TVFEMD-GWO-MOMEDA method can effectively filter the interference noise in the measured fault signal and effectively improve the significance of the fault characteristic frequency in the envelope spectrum. The results of the envelope spectrum analysis and evaluation index analysis show that the proposed method is better than the other four methods in extracting early inner-ring fault characteristics.

## 6. Conclusions

To solve the difficult challenge of extracting early fault features in bearings, this paper proposes a diagnostic method combining parameter-adaptive MOMEDA and TVFEMD. Based on the experiments on bearing fault simulation data and measured data, it can be concluded that this method can effectively enhance the periodic impact characteristics of early faults and has a good effect on bearing early fault diagnosis. The following conclusions can be drawn:

(1) By constructing a new composite index as the objective function of parameter optimization, the GWOMOMEDA method uses the excellent global search characteristics of the gray wolf optimization algorithm to determine the optimal influence parameters adaptively, which avoids the interference of human subjective factors in parameter selection and achieves optimal deconvolution results.
(2) The bearing early fault impact signal can easily cause modal aliasing, which is difficult to completely separate. The TVFEMD method overcomes the modal aliasing problem of the EMD method and can extract fault features from deconvolution signals well. The TVFEMD decomposition of the fault signal not only solves the problem of mode aliasing after the use of traditional EMD decomposition, but it also maintains the integrity of the fault signal better, which overcomes the shortcomings of other EMD improvement methods.
(3) The parameter-optimized MOMEDA method can significantly reduce the influence of noise on the TVFEMD method, and the effective modal components of the TVFEMD method are selected according to the weighted kurtosis index with higher reliability. Therefore, the combination of the parameter-optimized MOMEDA and TVFEMD methods can realize the accurate extraction of early fault features of rolling bearings, and the effect is better than the traditional MKCD deconvolution method and fast spectral kurtosis FSK class method.

In this paper, the key problems in rolling bearing fault diagnosis are studied and improved. Although some achievements have been made, further studies are needed in the following aspects: (1) This paper mainly focuses on the fault diagnosis of single bearing faults. Multipoint composite faults may occur in engineering practice, and it is necessary to study the composite fault diagnosis of rolling bearings in the future. (2) The diagnostic method proposed in this paper is conducted under constant speed and load conditions for fault determination. In practical engineering, due to the complex and variable operating conditions, the proposed method needs to be continuously improved in the future to make it more applicable to actual operating conditions.

## Figures and Tables

**Table 1:** Comparison of simulation signal analysis results.

Method	Evaluation Index			
	FCK/10^−4^	FFC/10^−2^	SE	ESE
FSK	0.93	1.09	1.483	1.117
MCKD	1.13	1.42	1.391	1.105
TVFEMD-MOMEDA(L = 600)	3.13	3.77	1.227	0.950
TVFEMD-MODEDA(Ts = 50)	2.16	2.95	1.275	0.986
TVFEMD-GWO-MOMEDA	5.52	6.14	1.124	0.846

**Table 2:** Parameters of the bearing.

Type	Inner Diameter	Outer Diameter	Pitch Diameter	Ball Diameter	Number of Balls	Angle
ER-12K	19.05 mm	47 mm	42.05 mm	7.94 mm	8	0

**Table 3:** Comparison of measured outer-race fault analysis results.

Method	Evaluation Index			
	FCK/10^−4^	FFC/10^−2^	SE	ESE
FSK	0.83	0.41	1.7149	1.2450
MCKD	1.94	1.02	1.7438	1.2755
TVFEMD-MOMEDA(L = 600)	2.97	2.29	1.5309	1.1509
TVFEMD-MODEDA(Ts = 50)	2.38	1.92	1.5644	1.1774
TVFEMD-GWO-MOMEDA	4.46	3.84	1.3392	1.0277

**Table 4:** Analysis results of comparison of the measured inner-race fault signal.

Method	Evaluation Index			
	FCK/10^−4^	FFC/10^−2^	SE	ESE
FSK	1.37	1.03	1.7726	1.3115
MCKD	1.92	1.65	1.7953	1.3290
TVFEMD-MOMEDA(L = 600)	3.01	2.98	1.5861	1.1894
TVFEMD-MODEDA(Ts = 50)	2.77	2.29	1.5763	1.1979
TVFEMD-GWO-MOMEDA	4.79	4.26	1.4926	1.0752

## Data Availability

Available online: http://ides.nuaa.edu.cn.

## Nomenclature

EMD	empirical mode decomposition
TVFEMD	time-varying filtering EMD
MOMEDA	multipoint optimal minimum entropy deconvolution adjusted
IMF	intrinsic mode function
EEMD	ensemble empirical mode decomposition
CEEMD	complementary ensemble empirical mode decomposition
CEEMDAN	complete ensemble empirical mode decomposition with adaptive noise
ESK	envelope spectrum kurtosis
MOAF	maximum of autocorrelation function
GWO	gray wolf optimizer
FSK	fast spectral kurtosis
MCKD	maximum correlation kurtosis deconvolution
FCK	first-order correlated kurtosis
FFC	fault feature coefficient
SE	sampling entropy
ESE	envelope spectrum entropy
